# Impact of comorbid constipation on the survival of patients with heart failure: a multicenter, prospective cohort study conducted in Japan

**DOI:** 10.3389/fcvm.2024.1470216

**Published:** 2025-01-14

**Authors:** Tomoaki Ishida, Kei Kawada, Kohei Jobu, Tomoyuki Hamada, Toru Kubo, Moemi Okazaki, Kazuya Kawai, Yoko Nakaoka, Toshikazu Yabe, Takashi Furuno, Eisuke Yamada, Hiroaki Kitaoka, Yukihiro Hamada

**Affiliations:** ^1^ Department of Pharmacy, Kochi Medical School Hospital, Nankoku, Japan; ^2^ Department of Clinical Pharmacy Practice Pedagogy, Tokushima University Graduate School of Biomedical Sciences, Tokushima, Japan; ^3^ Department of Clinical Pharmacology and Therapeutics, Tokushima University Graduate School of Biomedical Sciences, Tokushima, Japan; ^4^ Department of Cardiology and Geriatrics, Kochi Medical School, Kochi University, Nankoku, Japan; ^5^ Department of Cardiology, Chikamori Hospital, Kochi, Japan; ^6^ Department of Cardiology, Kochi Prefectural Hatakenmin Hospital, Sukumo, Japan; ^7^ Department of Cardiology, Kochi Prefectural Aki General Hospital, Aki, Japan; ^8^ Department of Cardiology, Susaki Kuroshio Hospital, Susaki, Japan

**Keywords:** heart failure, constipation, risk factors, propensity score matching, mortality

## Abstract

**Background:**

Constipation frequently affects heart failure patients because of medication side effects and physiological effects of the condition. Although recent speculation suggests that comorbid constipation may affect cardiovascular disease onset and survival rates, this relationship remains unclear. We examined the effect of comorbid constipation on the survival of patients with heart failure.

**Methods:**

We conducted a multicenter prospective cohort study (the Kochi YOSACOI study) of patients hospitalized for acute decompensated heart failure. The influence of comorbid constipation on survival was evaluated using Cox regression analysis with 2-year survival as the index. Patients were divided into two groups based on the presence of comorbid constipation. The patient background was adjusted using propensity score matching, and the evaluation included assessing the 2-year survival and cardiovascular mortality occurrence using the log-rank test.

**Results:**

Among 1,061 patients hospitalized for acute decompensated heart failure, 715 with complete data (124 with comorbid constipation and 591 without) were analyzed. Comorbid constipation was identified as a risk factor for poorer survival in the Cox regression model (hazard ratio: 1.90, 95% confidence interval: 1.3–2.8, *P* < 0.001). Propensity score matching included 104 patients in each group. Survival analysis using the log-rank test indicated worse survival (*P* = 0.023) and higher cardiovascular mortality (*P* = 0.043) in the comorbid constipation group.

**Conclusion:**

Constipation can negatively affect the survival of patients with heart failure. Although the causal link between constipation and decreased survival remains unclear, identifying comorbid constipation is essential for identifying heart failure patients at a higher risk of poor outcomes.

## 1 Introduction

Cardiovascular disease is the leading cause of death globally, with heart failure (HF) being a serious condition characterized by high incidence and mortality rates (1). HF progresses through a series of exacerbations and remissions (2). The cost of treatment and diminishing quality of life (QOL) as the disease advances impose a significant economic burden on patients, families, and society (3). Therefore, to improve treatment and care for patients with HF, it is crucial to understand the factors associated with worsening prognosis. This understanding may help to identify the causes of rehospitalization and death (3). Several factors influence the prognosis of patients with HF. Heart-related factors, such as the left ventricular ejection fraction and atrial fibrillation, are known contributors (4, 5). Additionally, underlying conditions, such as diabetes, dyslipidemia, and hypertension, which are causes of cardiac diseases, play significant roles (6–8). Numerous studies have documented these factors, and international guidelines have emphasized the importance of adopting measures for patients with such comorbidities (6–9). Given that HF commonly affects older adults and is frequently associated with multiple underlying diseases (10), further research on comorbidities and their impact on HF is warranted.

Constipation usually presents with a decreased frequency of bowel movements and residual stools and is caused by organ-related factors, diet, lifestyle, and side effects of drugs (11). A Japanese study reported that 3.5% of the population suffers from constipation, with its prevalence worsening with age. Among individuals over 80 years old, the prevalence increases to 10% (12, 13). In developed countries, the population of patients with HF is aging, especially in Japan, which has the highest proportion of older adults aged 65 and over due to its long life expectancy and low birth rate (14). As a result, the average age of patients with HF in Japan is 79 years, which is higher than in other countries. This population is more likely to be affected by age-related diseases compared to patients in countries like the United States (average age 73 years) and Taiwan (average age 74 years), both of which are also developed countries (15). Moreover, constipation is more prevalent among patients with cardiovascular disease. A previous study in Japan reported a higher prevalence of 47% of constipation among patients hospitalized for cardiovascular conditions (16). Additionally, 33% of older adult patients with HF in the United States (aged 65 and older) reported a history of constipation (17). Recent research has shown that patients with comorbid constipation are at an increased risk of developing cardiovascular diseases such as coronary artery disease and ischemic stroke (16, 17). Furthermore, constipation has been linked to higher rates of rehospitalization among patients with HF (13). However, no studies have yet examined whether constipation impacts mortality in patients with HF. Therefore, this study aims to investigate the effect of constipation on mortality in patients with HF in Japan, where the older adult population is particularly high, to emphasize the importance of managing bowel movements as part of HF treatment.

## 2 Materials and methods

### 2.1 Patient population

This study used data from the Kochi Registry of Subjects with Acute Decompensated HF (YOSACOI) study, which registered 1,061 patients hospitalized for acute decompensated HF in Kochi, Japan, between May 2017 and December 2019. The dataset for this study was accessed on April 20, 2023. Patients were followed up until December 2021 to gather data on clinical outcomes, specifically focusing on all-cause mortality within a 2-year period. The details of the YOSACOI study have been explained in previous studies (18). The YOSACOI study involved collaboration among six hospitals that provide acute care for cardiovascular diseases in Kochi Prefecture, where the population of older adults aged ≥65 years constituted 35%. All participating hospitals undertook acute HF treatment according to standard guidelines (19). The eligibility criteria for enrolment were age ≥20 years and admission for acute decompensated HF (ADHF) at one of the participating hospitals. According to the Framingham criteria, ADHF is diagnosed based on the presence of at least two major criteria, including symptoms, physical examination, chest radiography, and echocardiographic findings, or one major and two minor criteria. Comorbid constipation was defined as the continuous use of laxatives from pre-admission to discharge.

This study was approved by the Ethics Committee of the Kochi Medical School (Approval No. 28–68). The study conformed to the principles outlined in the Declaration of Helsinki, and written informed consent was obtained from all patients or their families. Confidentiality and anonymity of patient data were maintained throughout.

### 2.2 Study design and statistical analysis

The researchers collected data from the participating hospitals during the enrolment period. We obtained information regarding patient demographics, HF etiology, medical history, HF symptoms, vital signs at discharge, laboratory data, echocardiographic data, other clinical parameters, and discharge prescriptions. The patients' nutritional status was assessed using the Geriatric Nutritional Risk Index (GNRI), a simple indicator of nutritional status in older adults, calculated using the following formula: GNRI = 14.89 × serum albumin (g/dl) + 41.7 × body weight (kg)/ideal body weight (kg) (20). Renin-angiotensin system (RAS) inhibitor use was defined as the prescription of an angiotensin-converting enzyme inhibitor or angiotensin-II receptor antagonist. Angiotensin receptor neprilysin inhibitors and sodium-glucose cotransporter-2 inhibitors were not approved in Japan during the registration period (21).

**Figure 1:**
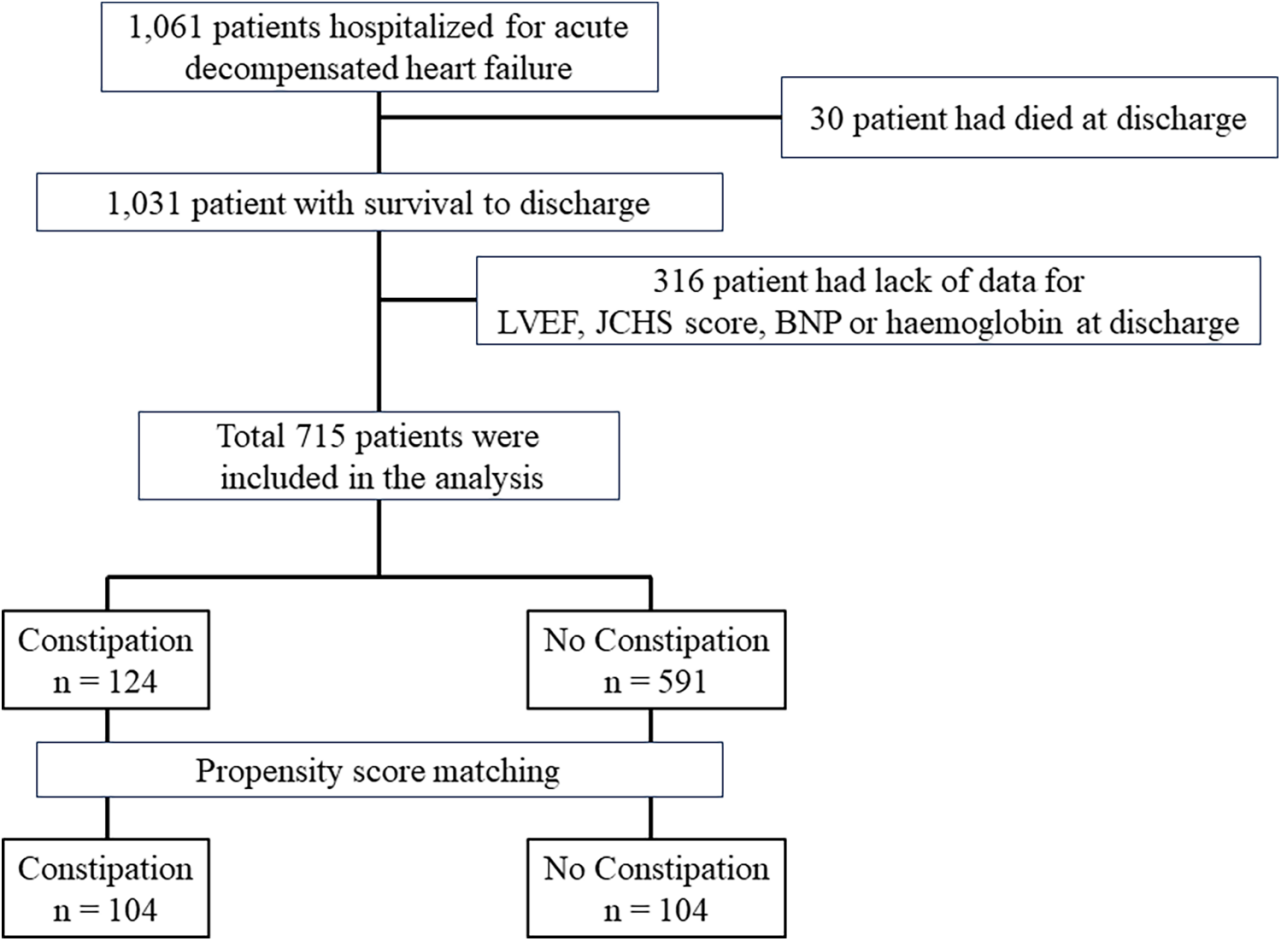
Study flow chart.

A flowchart depicting the patient selection process is shown in Figure 1. Among the initial patient cohort (*n* = 1,061), 30 patients who were discharged owing to death, and 316 patients with missing data on left ventricular ejection fraction (LVEF), the Japanese version of the Cardiovascular Health Study (J-CHS) score, brain natriuretic peptide (BNP) level, and medications were excluded. The remaining 715 cases were analyzed in this study. First, the 715 patients were divided into two groups based on whether they died within 2 years of enrolment, and the patient backgrounds of each group were compared. Second, univariate and multivariate Cox regression analyses were performed to evaluate the adjusted risk ratios of the variables in 715 patients included in the analysis. Factors with *P* < 0.1 on univariate analysis were used as explanatory variables for multivariate Cox regression analysis. The number of events per variable in the Cox regression analyses was set according to previous studies (22), with an event-to-variable ratio of 9.7. Third, patients were divided into two groups based on the presence of comorbid constipation. Patient backgrounds were matched using propensity score matching. The factors used in propensity score matching were age, sex, GNRI, New York Heart Association (NYHA) class III/IV at discharge, systolic blood pressure (mmHg), hospitalization history, BNP, estimated glomerular filtration rate (eGFR), hemoglobin level, sodium level, LVEF, J-CHS score, comorbid atrial fibrillation, chronic obstructive pulmonary disease (COPD), diabetes, and the use of RAS inhibitors, β-blockers, mineralocorticoid receptor antagonists, loop diuretics, thiazide diuretics, and tolvaptan (8, 19, 21, 23–28). We selected these factors based on their reported associations with constipation and HF. After creating the propensity score, we conducted 1:1 nearest-neighbor matching of the logit of the propensity score using a caliper width of 0.2 standard deviations of the propensity score (29). Matching was performed without replacement, and cases that did not meet the matching criteria were excluded. Standardized differences were used to measure covariate balance, which was defined as an absolute value exceeding 1.96 × √2/*n* (30). After matching, 2-year mortality and mortality due to cardiovascular causes were compared using the log-rank test. The 2-year mortality rate for the non-constipation group was set at 81%, while the increased mortality rate due to constipation was set at 19%. Assuming a 1:1 composition for each group, with a two-sided alpha of 0.05% and 80% power, the required sample size for each group was 71 individuals.

Data are expressed as medians with interquartile ranges (IQR) for variables that are not normally distributed and as frequencies (percentages) for categorical variables. Differences in variables were analyzed using the Mann–Whitney *U* test. Fisher's exact test was used to analyze categorical data. Hazard ratios (HRs) and 95% confidence intervals (CIs) were calculated using Cox regression analysis, and multivariate Cox regression analysis was used to examine the adjusted relative risk of the variables. Propensity score matching and sample size calculation for the log-rank test were performed using R statistical software (R Foundation for Statistical Computing, Vienna, Austria). All other statistical analyses were conducted using EZR version 1.51 (Saitama Medical Center, Jichi Medical University, Saitama, Japan) (31).

## 3 Results

### 3.1 Univariate analysis of factors potentially associated with two-year mortality

Among the 715 patients, 155 died during the 2-year follow-up period. Patients were categorized based on 2-year mortality, and differences in patient characteristics between groups were analyzed using univariate analysis. The results (Table 1) showed that patients who died were older [median (IQR): 85 (80–90) vs. 80 (70–85) years, *P* < 0.001], had lower GNRI [median (IQR): 84.9 (73.0–93.0) vs. 89.5 (80.1–97.7), *P* < 0.001], lower frequency of NYHA class III/IV at discharge [8 (5.2%) vs. 10 (1.8%) cases, *P* = 0.036], lower frequency of prior hospitalization with HF [63 (41.2%) vs. 141 (25.3%) cases, *P* < 0.001], higher BNP [median (IQR): 362 (200–597) vs. 254 (131–468), *P* < 0.001], lower eGFR [median (IQR): 40.6 (25.9–52.8) vs. 46.0 (33.1–61.6), *P* < 0.001], lower sodium level [median (IQR): 138 (135–140) vs. 139 (137–141), *P* < 0.001], higher J-CHS score [median (IQR): 3 (3–4) vs. 2 (2–3), *P* < 0.001], higher frequency of constipation [45 (29.0%) vs. 79 (14.1%) cases, *P* < 0.001], higher frequency of COPD [27 (17.4%) vs. 41 (7.3%) cases, *P* = 0.001], and more frequent use of RAS inhibitors [57 (36.8%) vs. 294 (52.5%) cases, *P* = 0.001].

**Table 1:** Baseline characteristics of the patients based on death within 2 years.

	All patients	Not survived	Survived	*P* value
	(*n* = 715)	(*n* = 155)	(*n* = 560)	
Age (years)	81 (72–86)	85 (80–90)	80 (70–85)	<0.001
Female	349 (48.8)	72 (46.5)	277 (49.5)	0.32
BMI (kg/m^2^)	21.0 (18.8–23.3)	20.3 (17.4–22.5)	21.2 (19.1–23.6)	0.001
GNRI	91.3 (84.6–98.7)	84.9 (73.0–93.0)	89.5 (80.1–97.7)	<0.001
NYHA class III/IV at discharge	18 (2.5)	8 (5.2)	10 (1.8)	0.036
Prior heart failure hospitalization	204 (28.5)	63 (41.2)	141 (25.3)	<0.001
SBP (mmHg)	114 (100–126)	108 (97–124)	113 (101–126)	0.019
Laboratory data at discharge				
Albumin (g/dl)	3.5 (3.1–3.7)	3.3 (3.0–3.6)	3.5 (3.2–3.8)	<0.001
BNP (pg/ml)	276 (141–497)	362 (200–597)	254 (131–468)	<0.001
eGFR (ml/min/1.73 m^2^)	44.4 (32.1–60.0)	40.6 (25.9–52.8)	46.0 (33.1–61.6)	<0.001
Hemoglobin (g/dl)	11.5 (10.0–13.1)	10.9 (9.6–12.6)	11.6 (10.1–13.2)	<0.001
Sodium (mEq/L)	139 (137–141)	138 (135–140)	139 (137–141)	<0.001
Echocardiographic parameters				
LVEF (%)	48 (35–62)	46 (33–62)	49 (35–62)	0.35
≥45%	311 (43.5)	73 (47.1)	238 (42.5)	0.32
Frailty assessment				
J-CHS score	3 (2–3)	3 (3–4)	2 (2–3)	<0.001
1	99 (13.8)	11 (7.1)	88 (15.7)	0.005
2	199 (27.8)	26 (16.6)	173 (30.9)	<0.001
≥3	386 (54.0)	118 (76.1)	268 (47.9)	<0.001
Underlying disease				
Hypertension	537 (75.1)	114 (73.5)	423 (75.5)	0.60
Diabetes mellitus	214 (29.9)	41 (26.5)	137 (24.5)	0.60
Dyslipidemia	320 (44.8)	70 (45.2)	250 (44.6)	0.93
Constipation	124 (17.3)	45 (29.0)	79 (14.1)	<0.001
Atrial fibrillation	336 (47.0)	76 (49.0)	260 (46.4)	0.59
COPD	68 (9.5)	27 (17.4)	41 (7.3)	0.001
Medication				
RAS inhibitors	280 (39.2)	57 (36.8)	294 (52.5)	0.001
ACE inhibitor	163 (22.8)	22 (14.2)	141 (25.2)	0.003
Angiotensin-receptor blockers	189 (26.4)	35 (22.6)	154 (27.5)	0.26
β-blockers	444 (62.1)	83 (53.5)	360 (64.3)	0.53
MRAs	263 (36.8)	69 (44.5)	205 (36.6)	0.077
Loop diuretic	639 (89.4)	138 (89.0)	505 (90.2)	0.65
Thiazide diuretics	36 (5.0)	7 (10.0)	29 (4.5)	0.075
Tolvaptan	199 (27.8)	24 (34.3)	175 (27.1)	0.21

Data are presented as median (interquartile range) or *n* (%). Differences in variables were analyzed using the Mann-Whitney *U* test. In the case of categorical data, Fisher's exact test was used. ACE, angiotensin-converting enzyme; β-blocker, beta-adrenergic blocking agent; BMI, body mass index; BNP, brain natriuretic peptide; COPD, chronic obstructive pulmonary disease; eGFR, estimated glomerular filtration rate; GNRI, geriatric nutritional risk index; J-CHS, Japanese version of the cardiovascular health study; LVEF, left ventricular ejection fraction; MRAs, mineralocorticoid receptor antagonists; NYHA, New York heart association; RAS, renin-angiotensin system; SBP, systolic blood pressure.

### 3.2 Cox regression analysis of risk factors for all-cause mortality in 2 years

The results of multivariable Cox regression analysis, as shown in Table 2, revealed that underlying COPD (HR: 2.07, 95% CI: 1.28–3.35, *P* = 0.003), underlying constipation (HR: 1.86, 95% CI: 1.25–2.75, *P* = 0.002), higher J-CHS score (HR: 1.31, 95% CI: 1.12–1.54, *P* = 0.001), older age (HR: 1.06, 95% CI: 1.03–1.08, *P* < 0.001), and lower sodium level (HR: 0.90, 95% CI: 0.86–0.95, *P* < 0.001) were independently associated with all-cause mortality within 2 years (Table 2).

**Table 2:** Cox regression analysis of the risk factors for all-cause death (2 years) in patients with heart failure.

	Univariate HR	*P*-value	Multivariate HR	*P*-value
	(95% CI)		(95% CI)	
NYHA class III/IV at discharge	2.61 (1.28–5.32)	0.008	2.12 (0.95–4.72)	0.065
COPD	1.99 (1.30–3.05)	0.002	2.07 (1.28–3.35)	0.003
Constipation	2.22 (1.56–3.15)	<0.001	1.86 (1.25–2.75)	0.002
J-CHS score	1.55 (1.35–1.78)	<0.001	1.31 (1.12–1.54)	0.001
Prior heart failure hospitalization	1.92 (1.39–2.66)	<0.001	1.30 (0.89–1.90)	0.18
Tolvaptan	1.63 (1.17–2.26)	0.004	1.28 (0.87–1.87)	0.22
β-blockers	0.72 (0.53–0.99)	0.047	1.26 (0.87–1.82)	0.21
Thiazide diuretics	2.15 (1.31–3.52)	0.002	1.25 (0.72–2.19)	0.43
Age (years)	1.06 (1.04–1.08)	<0.001	1.06 (1.03–1.08)	<0.001
BNP (10^2^ pg/ml)	1.04 (1.01–1.07)	0.002	1.02 (0.99–1.05)	0.14
SBP (mmHg)	0.98 (0.97–0.99)	0.012	0.99 (0.98–1.00)	0.23
eGFR (ml/min/1.73 m^2^)	0.98 (0.98–0.99)	<0.001	0.99 (0.98–1.00)	0.19
Hemoglobin (g/dl)	0.86 (0.79–0.93)	<0.001	0.98 (0.89–1.08)	0.65
GNRI (10^2^)	0.44 (0.23–0.87)	0.018	0.92 (0.35–2.40)	0.86
Sodium (mEq/L)	0.88 (0.85–0.82)	<0.001	0.90 (0.86–0.95)	<0.001
RAS inhibitors	0.57 (0.41–0.79)	<0.001	0.80 (0.55–1.16)	0.23
MRAs	1.26 (0.92–1.74)	0.16		
Atrial fibrillation	1.13 (0.82–1.55)	0.46		
LVEF (%)	1.00 (0.99–1.01)	0.67		
Dyslipidemia	0.96 (0.70–1.32)	0.81		
Female	0.95 (0.69–1.30)	0.75		
Loop diuretic	0.87 (0.53–1.44)	0.59		
Diabetes mellitus	0.84 (0.58–1.19)	0.32		

Data are presented as median [interquartile range) or *n* (%). Hazard ratios (HRs) and 95% confidence intervals (CIs) were determined using COX hazard regression analysis, and multivariate Cox hazard regression analysis was used to analyze the adjusted relative risk of variables. β-blocker, beta-adrenergic blocking agent; BNP, brain natriuretic peptide; COPD, chronic obstructive pulmonary disease; eGFR, estimated glomerular filtration rate; GNRI, geriatric nutritional risk index; J-CHS, Japanese version of the cardiovascular health study; LVEF, left ventricular ejection fraction; MRAs, mineralocorticoid receptor antagonists; NYHA, New York heart association; RAS, renin-angiotensin system; SBP, systolic blood pressure.

### 3.3 Baseline characteristics of the patients based on underlying constipation

**Figure 2:**
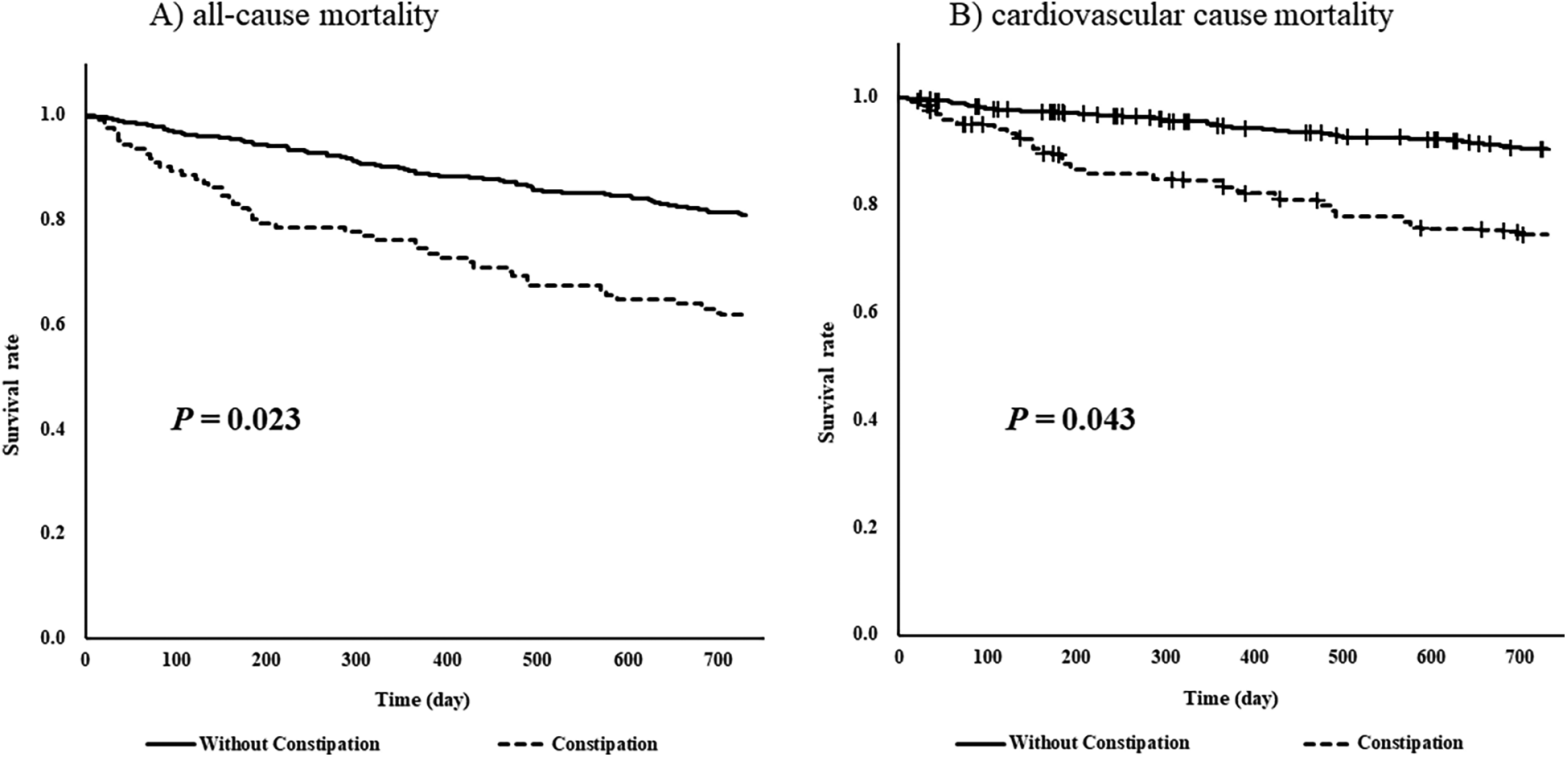
Log-rank tests of **(A)** all-cause mortality and **(B)** cardiovascular cause mortality for 730 days (2 years) by underlying constipation stratified by propensity-score matching.

After 1:1 matching, equal numbers of patients with underlying constipation (*n* = 104 per group) were matched, and the cut-off value of the absolute standardized difference for imbalance was 0.27. Across the baseline covariates, the absolute standardized differences ranged from 0.003–0.22, indicating that the mean and frequency of continuous and dichotomous variables were similar in both matched groups (Table 3). Log-rank tests showed that the group with comorbid constipation had a higher incidence of all-cause mortality (*P* = 0.023) and cardiovascular death (*P* = 0.043) than the non-comorbid constipation group (Figure 2).

**Table 3:** Baseline characteristics before and after propensity-score matching.

	Before propensity-score matching			After propensity-score matching			
	Constipation	Without constipation	*P* value	Constipation	Without constipation	*P* value	Standardized difference
	(*n* = 124)	(*n* = 591)		(*n* = 104)	(*n* = 104)		
Age (years)	84 (78–89)	81 (71–86)	<0.001	84 (78–89)	84 (81–89)	0.71	0.040
Female	60 (48.4)	289 (48.9)	0.92	53 (51.0)	51 (49.0)	0.89	0.048
BMI (kg/m^2^)	20.7 (18.5–23.0)	21.1 (18.9–23.6)	0.29	20.6 (18.4–22.7)	21.0 (18.4–22.4)	0.98	0.032
GNRI	88.0 (78.8–97.0)	88.7 (78.1–96.9)	0.69	89.6 (83.0–96.7)	88.8 (81.6–95.4)	71	0.027
NYHA class III/IV at discharge	1 (0.8)	17 (2.9)	0.34	1 (1.0)	2 (1.9)	0.99	0.080
Prior heart failure hospitalization	51 (41.1)	153 (26.1)	0.001	39 (37.5)	41 (39.4)	0.89	0.032
SBP (mmHg)	112 (98–129)	112 (101–125)	0.76	112 (98–128)	111 (99–125)	0.99	0.019
Laboratory data at discharge							
Albumin (g/dl)	3.5 (3.1–3.7)	3.5 (3.1–3.7)	0.95	3.4 (3.1–3.8)	3.4 (3.0–3.7)	0.20	0.22
BNP (pg/ml)	250 (168–465)	284 (138–507)	0.52	250 (167–469)	290 (149–493)	0.52	0.16
eGFR (ml/min/1.73 m^2^)	40.0 (30.6–53.8)	45.2 (32.4–60.9)	0.041	39.9 (31.6–53.8)	44.0 (32.4–58.5)	0.58	0.021
Hemoglobin (g/dl)	11.4 (9.9–12.5)	11.5 (10.1–13.2)	0.135	11.3 (9.9–12.5)	10.9 (10.0–12.2)	0.63	0.071
Sodium (mEq/L)	138 (136–140)	139 (137–141)	0.039	139 (137–141)	139 (137–141)	0.57	0.028
Echocardiographic parameters							
LVEF (%)	49.0 (36.3–64.0)	48.0 (34.0–62.0)	0.28	49 (36–64)	52 (37–64)	0.79	0.015
≥45%	76 (61.3)	340 (57.5)	0.48	63 (60.6)	66 (63.5)	0.78	0.067
Frailty assessment							
J-CHS score	3 (2–4)	3 (2–3)	0.009	3 (2–3)	3 (2–4)	0.51	0.082
1	11 (8.9)	88 (14.9)	0.086	10 (9.6)	12 (11.5)	0.82	0.093
2	33 (26.6)	166 (28.1)	0.83	29 (27.9)	23 (22.1)	0.42	0.14
≥3	78 (62.9)	308 (52.1)	0.03	63 (60.6)	66 (63.5)	0.78	0.047
Underlying disease							
Hypertension	95 (76.6)	442 (74.8)	0.73	80 (76.9)	78 (75.0)	0.87	0.063
Diabetes mellitus	40 (32.3)	174 (29.4)	0.59	35 (33.7)	30 (28.8)	0.55	0.11
Dyslipidaemia	56 (45.2)	264 (44.7)	0.92	47 (45.2)	47 (45.2)	0.99	0.009
Atrial fibrillation	63 (50.8)	273 (46.2)	0.37	50 (48.1)	53 (51.0)	0.78	0.048
COPD	15 (12.1)	53 (9.0)	0.31	12 (11.5)	14 (13.5)	0.83	0.085
Medication							
RAS inhibitors	34 (27.4)	317 (53.6)	<0.001	28 (26.9)	25 (24.0)	0.76	0.073
ACE inhibitor	15 (12.1)	148 (25.0)	0.001	13 (12.5)	15 (14.4)	0.84	0.082
Angiotensin-receptor blockers	19 (15.3)	169 (28.6)	0.002	15 (14.4)	10 (9.6)	0.39	0.15
β-blockers	61 (49.2)	383 (64.8)	0.002	51 (49.0)	60 (57.7)	0.89	0.18
MRAs	48 (38.7)	226 (38.2)	0.92	40 (38.5)	50 (48.1)	0.21	0.21
Loop diuretic	118 (95.2)	525 (88.8)	0.033	98 (94.2)	100 (96.2)	0.75	0.092
Thiazide diuretics	10 (8.1)	26 (4.4)	0.11	9 (8.7)	9 (8.7)	0.99	0.003
Tolvaptan	50 (40.3)	149 (25.2)	<0.001	38 (36.5)	38 (36.5)	0.99	0.007

Data are presented as median (interquartile range) or *n* (%). Data are presented as median (interquartile range) or *n* (%). Differences in variables were analyzed using the Mann-Whitney *U* test. In the case of categorical data, Fisher's exact test was used. ACE, angiotensin-converting enzyme; β-blocker, beta-adrenergic blocking agent; BMI, body mass index; BNP, brain natriuretic peptide; COPD, chronic obstructive pulmonary disease; eGFR, estimated glomerular filtration rate; GNRI, geriatric nutritional risk index; J-CHS, Japanese version of the cardiovascular health study; LVEF, left ventricular ejection fraction; MRAs, mineralocorticoid receptor antagonists; NYHA, New York heart association; RAS, renin-angiotensin system; SBP, systolic blood pressure.

### 3.4 Discussion

To the best of our knowledge, this is the first study to investigate the effect of constipation on the prognosis of patients with HF. The results of Cox regression analysis suggested that constipation may increase the risk of all-cause mortality in patients with HF. Furthermore, when life expectancy was compared using propensity score matching after dividing patients according to constipation, patients with HF and constipation showed a higher incidence of all-cause and cardiovascular mortality rates.

Constipation is a prevalent condition (32). Historically, constipation was regarded as a benign, non-life-threatening condition (33). However, recent studies have linked constipation to an increased risk of mortality (17). Furthermore, underlying constipation may cause complications or worsen the comorbidities (16, 34). Observational studies have indicated a higher likelihood of cardiovascular events such as stroke and myocardial infarction in individuals with underlying constipation (16, 35). This finding suggests a potential impact on the occurrence or exacerbation of other cardiovascular diseases, including HF. Nevertheless, no study has specifically examined the influence of a history of constipation on the prognosis of patients with HF. Therefore, our study aimed to investigate the association between constipation and prognosis in patients with HF. Cox regression analysis revealed that a history of constipation was a risk factor for early death. Furthermore, to examine the impact of constipation on the prognosis of patients with HF, we divided patients into groups based on the presence of constipation, matched patient backgrounds using propensity score matching, and evaluated the survival rate. Our results suggest that underlying constipation may significantly increase all-cause and cardiovascular mortality rates.

Several hypotheses have been proposed to explain the mechanisms by which constipation worsens symptoms of HF. First, straining during defecation can elevate blood pressure, potentially triggering cardiovascular events such as arrhythmias, acute coronary events, and aortic dissection (36). Elevated blood pressure also imposes an increased cardiac workload on patients with HF, potentially worsening their prognosis.

Second, changes in intestinal flora due to constipation can lead to an increase in inflammatory mediators (16). In patients with HF, hypoperfusion of the viscera leads to intestinal ischemia and edema, which has been proposed as a hypothetical mechanism by which the intestinal barrier function is compromised, and bacteria and their metabolites enter the systemic circulation (37). This process triggers both local and systemic inflammatory responses. Furthermore, gut microbiota contributes to endothelial dysfunction by promoting the upregulation of inflammatory signaling and the migration of leukocytes to endothelial cells through metabolites such as trimethylamine and uremic toxins (38–40). Endothelial dysfunction is a central factor in the pathogenesis of various cardiac disorders, including HF. It induces oxidative stress, endothelial impairment, increased secretion of adipokines, inflammatory cytokines (e.g., IL-6, TNF-α), endothelin-1, and fibroblast growth factors, as well as reduced nitric oxide production (41). These alterations in inflammatory cytokines exacerbate cardiac metabolism and diastolic function, thereby promoting the development and progression of cardiovascular diseases, including HF, and worsening their symptoms (41, 42). Therefore, if constipation negatively impacts the pathogenesis of HF, early intervention for constipation—such as pharmacotherapy, diet and exercise, and appropriate bowel management—may improve the prognosis of HF.

Constipation is common in patients with HF, and several hypotheses have been proposed to explain its prevalence in this population. First, gastrointestinal dysmotility resulting from an imbalance between sympathetic and parasympathetic activity plays a significant role (43). Increased sympathetic nervous system activity, which is characteristic of HF, reduces visceral circulation and, combined with a decreased cardiac output, can lead to intestinal ischemia. Studies have shown that blood flow to the intestines in patients with HF is reduced by approximately 30%–40% (44), impairing intestinal motility and contributing to constipation. In addition, this imbalance is also evident in HF, where heightened sympathetic nervous system activity places additional strain on the heart (45). Additionally, the high prevalence of constipation in patients with HF can be attributed to the aging population, as HF is more common among the older adults. Aging is a known risk factor for both HF and constipation, with physiological changes such as decreased gastrointestinal transit time and altered gut microbiota composition (46). In this study, the median age of patients with constipation was approximately 84 years, significantly older than that of patients without constipation [median (IQR): 84 (78–89) vs. 81 (71–86) years, *P* < 0.001]. This finding is consistent with previous observational studies (13). Another contributing factor is the reduction in total body water due to fluid restriction, a common management strategy in HF treatment (47). Diuretics, which are frequently prescribed to patients with HF, can decrease stool moisture content, exacerbating constipation (48). In this study, a higher proportion of constipated patients were prescribed diuretics [Loop diuretics: 118 (95.2%) vs. 525 (88.8%), *P* = 0.033; Tolvaptan: 50 (40.3%) vs. 149 (25.2%), *P* < 0.001], suggesting that diuretic use is a significant contributor to constipation in this population. Furthermore, intestinal permeability abnormalities resulting from hypoperfusion may lead to systemic inflammation, which can influence both HF progression and gastrointestinal function. These interconnected factors suggest a bidirectional relationship where constipation may exacerbate HF symptoms and vice versa. However, after analyzing patient backgrounds, including age and diuretic use, through propensity score matching, a higher mortality rate was observed among patients with constipation. This suggests that constipation may be an independent risk factor for increased mortality in patients with HF beyond the effects of diuretic use and other confounders.

### 3.5 Limitations

This study had a few limitations. First, it was an observational study. Although covariates were adjusted using propensity score matching to minimize selection bias, the findings should be interpreted with caution due to potential residual confounding and imbalances in baseline patient characteristics. Furthermore, propensity score matching cannot completely exclude all confounders, which may introduce selection bias. Therefore, the results must be verified in larger prospective studies or RCTs to ensure their validity and generalizability. Second, the symptoms associated with constipation were not comprehensively assessed in this observational study. Constipation was defined based on the use of constipation medication; therefore, future studies requiring evaluation of symptoms such as constipation severity should consider defining constipation according to symptom criteria such as the Rome III Criteria or other relevant standards (49). Third, the effect of the choice of constipation medication on the prognosis of HF has not yet been clarified. In this study, patients with constipation were administered different laxatives. Magnesium oxide was the most commonly prescribed agent (Supplementary Figure S1). However, hypermagnesemia reportedly negatively affects the prognosis of HF (50). Future studies should clarify the laxatives that are appropriate for use in patients with HF. Fourth, this study was unable to examine the patient's background regarding physical activity levels and dietary habits. These factors could have influenced both constipation and HF severity (51, 52). The absence of these confounding variables may affect the precision of our findings. Incorporating them into the analysis as confounding factors would have allowed for a more accurate examination of the relationship between constipation and HF outcomes. Future research should consider including physical activity and dietary assessments to enhance the validity of the results. Fifth, this study did not evaluate the severity of constipation. The severity of constipation, including factors such as frequency of defecation and duration of constipation episodes, may significantly influence HF outcomes and other comorbidities (53). Incorporating standardized measures of constipation severity into the analysis would enable a more precise assessment of its impact on HF prognosis. Future research should consider employing validated tools to grade constipation severity, thereby allowing for more accurate interclass comparisons and reducing potential variability among patients. Sixth, this study did not investigate the effect of constipation on quality of life (QOL). Previous studies have suggested that individuals with chronic constipation may experience reduced mental, social, and physical aspects of QOL compared with healthy individuals (33). However, the impact of constipation on QOL, specifically among patients with HF, has not been explored in this study. The absence of QOL assessment may limit the comprehensive understanding of how constipation affects overall patient well-being and disease management in HF. Therefore, future studies should incorporate validated QOL measurement tools to investigate this relationship and clarify the broader implications of constipation on the quality of life in HF populations.

In conclusion, this study suggests that constipation may negatively impact the prognosis of patients with HF. Although the causal relationship between constipation and worse prognosis in patients with heart failure is unknown, the presence of comorbid constipation should be considered when identifying patients with heart failure and poor prognosis.

## Data Availability

The raw data supporting the conclusions of this article will be made available by the authors, without undue reservation.
