# Supplementary material for: Impact of comorbid constipation on the survival of patients with heart failure: a multicenter, prospective cohort study conducted in Japan
Source: Front Cardiovasc Med. 2025 Jan 14;11:1470216. doi: 10.3389/fcvm.2024.1470216 (PMC11772488; doi:10.3389/fcvm.2024.1470216)
Supplement: Supplementary file 1 [file Presentation1.pptx]

## Slide 1
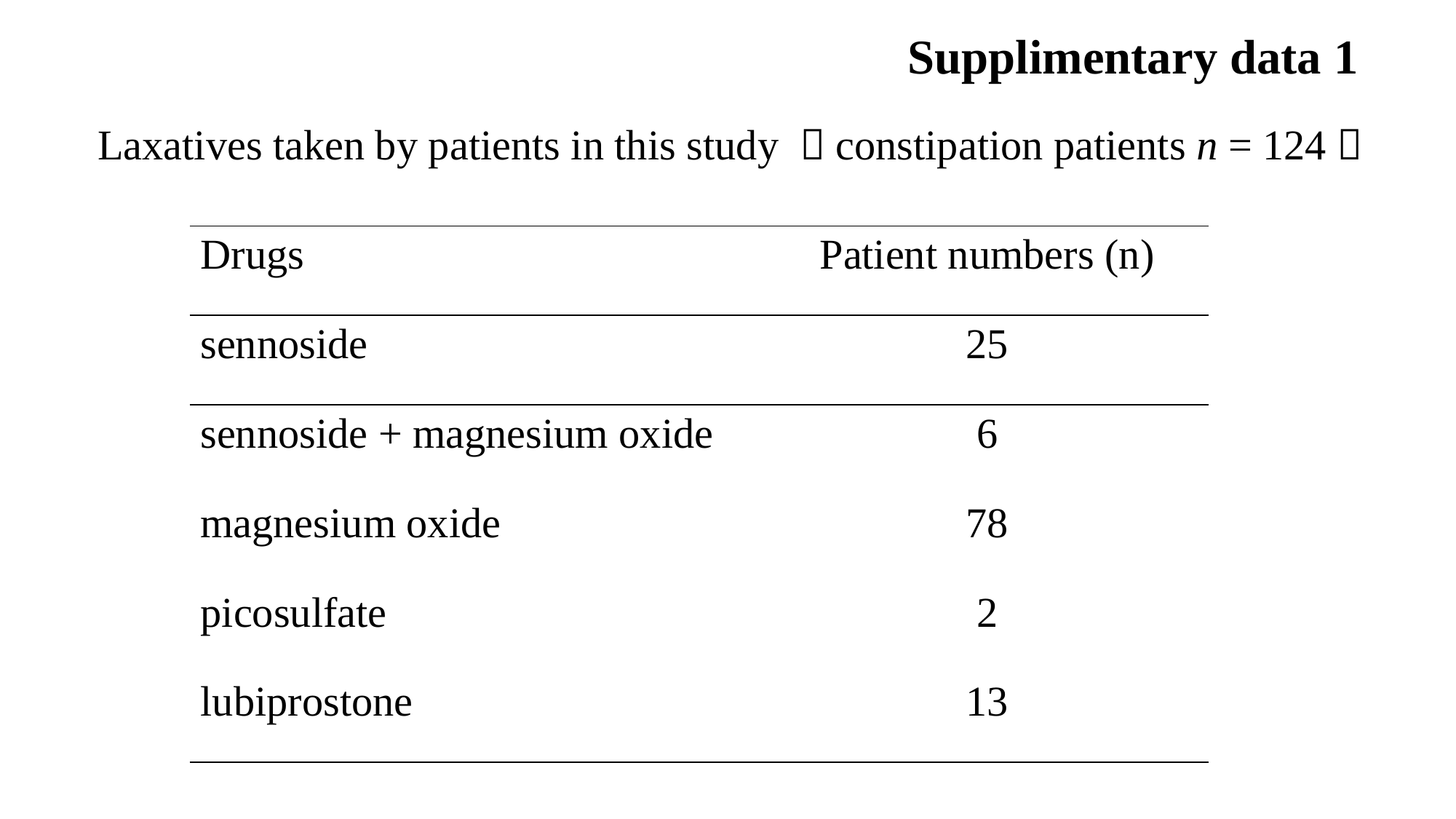

Supplimentary data 1
Laxatives taken by patients in this study （constipation patients n = 124）
| Drugs | Patient numbers (n) |
| --- | --- |
| sennoside | 25 |
| sennoside + magnesium oxide | 6 |
| magnesium oxide | 78 |
| picosulfate | 2 |
| lubiprostone | 13 |
